# Mild Caloric Restriction Decreases Insulin Requirements in Patients With Type 2 Diabetes and Severe Insulin Resistance

**DOI:** 10.1097/MD.0000000000001160

**Published:** 2015-07-31

**Authors:** Cristina Adelia Meehan, Elaine Cochran, Megan Mattingly, Phillip Gorden, Rebecca J. Brown

**Affiliations:** From the Diabetes, Endocrinology, and Obesity Branch, National Institute of Diabetes and Digestive Kidney Diseases, National Institutes of Health, Bethesda, Maryland.

## Abstract

Type 2 diabetes (T2D) affects ∼10% of the US population, a subset of whom have severe insulin resistance (SIR) (>200 units/d). Treatment of these patients with high-dose insulin presents logistical and compliance challenges. We hypothesized that mild caloric restriction would reduce insulin requirements in patients with T2D and SIR.

This was a retrospective study at the National Institutes of Health Clinical Center. Inclusion criteria were as follows: T2D, and insulin dose >200 units/d or >2 units/kg/d. The intervention consisted of mild caloric restriction during a 3 to 6-day hospitalization. The major outcomes were change in insulin dose and blood glucose from admission to discharge.

Ten patients met inclusion criteria. Baseline glycated hemoglobin A_1c_ was 10.0 ± 1.6% and body mass index 38.8 ± 9.0 kg/m^2^. Food intake was restricted from 2210 ± 371 kcal/d preadmission to 1810 ± 202 during the hospital stay (16.5% reduction). Insulin dose decreased from 486 ± 291 units/d preadmission to 223 ± 127 at discharge (44% reduction, *P* = 0.0025). Blood sugars decreased nonsignificantly in the fasting state (from 184 ± 85 to 141 ± 42, *P* = 0.20), before lunch (239 ± 68 to 180 ± 76, *P* = 0.057), and at bedtime (212 ± 95 to 176 ± 48, *P* = 0.19), and significantly decreased before dinner (222 ± 92 to 162 ± 70, *P* = 0.016).

Mild caloric restriction, an accessible and affordable intervention, substantially reduced insulin doses in patients with T2D and SIR. Further studies are needed to determine if the intervention and results are sustainable outside of a hospital setting.

## INTRODUCTION

Insulin resistance occurs on a spectrum of severity, ranging from common, milder forms associated with obesity, type 2 diabetes (T2D), and polycystic ovarian syndrome to much more severe, rare syndromic forms of insulin resistance, including insulin receptor mutations, autoantibodies to the insulin receptor, and lipodystrophies.^5^ A minority of patients with nonsyndromic T2D meet criteria for severe insulin resistance (SIR), which is defined as either extreme endogenous production of insulin, or high exogenous insulin requirements, either >2 to 3 units per kg body weight per day, or >200 units of insulin per day.^2^ In the context of growing numbers of patients with T2D and extreme obesity, the management of SIR has become a major clinical challenge.^2^ Effective treatment of patients with SIR is necessary to avoid micro- and macrovascular complications of diabetes.^2^

The treatment of SIR is managed most effectively with U-500 insulin therapy. Because U-500 is 5 times more concentrated than U-100, patients can inject smaller volumes, which both increases patient compliance and enhances efficacy by improving the insulin absorption rate.^8^ In addition, due to the longer half-life of U-500 compared with U-100 regular human insulin, patients are able to decrease daily insulin injections from 5 to 8 per day with U-100 to 2 to 3 per day with U-500.^4^

Diet is a key component to managing both syndromic and nonsyndromic forms of SIR. Severe caloric restriction from either very low calorie diets (VLCDs) of <800 kcal/d or bariatric surgery allows for rapid restoration of insulin sensitivity, independent of weight loss.^1,6,7,9,11^ However, VLCDs are not typically sustainable, and many patients with SIR are not good candidates for bariatric surgery; thus, alternative treatments are needed.

In the current study, we hypothesized that a sustainable moderate caloric restriction could significantly reduce insulin requirements in patients with nonsyndromic SIR. If correct, this would suggest that a small lifestyle change could lead to substantial clinical benefits.

## SUBJECTS AND METHODS

The present study was a retrospective chart review of patients enrolled in a natural history study of insulin resistance at the Clinical Center of the National Institutes of Health (NIH). The study was approved by the Institutional Review Board of the National Institute of Diabetes and Digestive and Kidney Diseases and was registered at clinicaltrials.gov (NCT00001987). All patients provided written informed consent after full explanation of the purpose and nature of all procedures used. Patients were referred to the research group by local physicians seeking better treatment for their patients’ high insulin requirements. Inclusion criteria for the current analysis were as follows: nonsyndromic T2D, insulin requirement of >200 units/d or >2 units/kg/d, and admission between 2005 and 2014.^2^ Patients were ineligible for the study if they had impaired kidney function defined by creatinine levels above 1.3 mg/dL, were using corticosteroids, or had a recent history of noncompliance with insulin therapy.

Patients were admitted to the NIH Clinical Center for 3 to 6 days and given an intervention consisting of a moderately calorically restricted diet (range 1600–2200 kcal/d) consisting of 50% carbohydrate, 20% protein, and 30% fat. The primary outcome of the study was the change in total daily insulin dose from the preadmission dose to the time of discharge. Secondary outcomes included change in insulin dose over time and capillary blood glucose levels. During the hospital visit capillary blood glucoses were measured each day at 4 time points: fasting, lunch, dinner, and bedtime using a hospital bedside glucometer (coefficient of variation ≤10%) and as needed for symptomatic hypoglycemia. Laboratory data, capillary blood glucose, and insulin doses were extracted from the NIH Clinical Center electronic medical record system. Preadmission insulin doses were calculated by patient self-report for fixed dose insulin regimens, and estimated using average blood glucose (based on glycated hemoglobin A_1c_ [HbA_1c_]) for patients using sliding scale insulin dosing. Subjects who were deemed noncompliant with reported home insulin dosing based on investigator judgment were excluded from the cohort.

Preadmission caloric intake was estimated based on admission body weight, assuming that patients were weight stable. Owing to inaccuracy of patient self-report of diet, the preadmission caloric intake was defined as the caloric intake needed to maintenance admission body weight. The Mifflin–St. Jeor equation was used with an activity factor of 1.3 (sedentary to light activity) to estimate calories for weight maintenance (Mifflin–St. Jeor equation Male: basal metabolic rate = 9.9 × weight (kg) + 6.25 × height (cm) − 5 × age (years) + 5, Female: 9.9 × weight (kg) + 6.25 × height (cm) − 5 × age (years) − 161).^10^

Insulin doses were reduced upon admission to minimize the risk of hypoglycemia based on empirical data on the effects of hospitalization on insulin requirements in patients with SIR.^3^

Statistical analyses were performed using GraphPad Prism version 6.01 for Windows (La Jolla, CA) and SAS Enterprise Guide 5.1 (Cary, NC). Paired *t* tests or Wilcoxon matched-pairs tests were used for comparisons between baseline and end-of-study values. For repeated measures analysis over multiple study days, mixed models (PROC MIXED) were used. Results are presented as mean ± SD (range) unless indicated otherwise. A *P* value <0.05 was considered statistically significant.

## RESULTS

Fourteen patients with T2D and SIR were admitted for caloric restriction. Four patients were excluded: 2 because their insulin requirements were <2 units/kg/d, 1 because consent was withdrawn, and the fourth because there was history of cirrhosis and severe hypoglycemia. Baseline characteristics of the 10 included patients are given in Table 1. Metabolic status was defined by baseline HbA_1c_ 10.0 ± 2 (8.4–13)% and body mass index (BMI) 38.8 ± 9 (29–55) kg/m^2^. Patients had a mean age of 51.3 ± 9 (35–65) years with diabetes duration of 11.4 ± 9 (3.0–25) years and had used insulin for 5.9 ± 5 (1.5–17) years. In addition to the metabolic status data in Table 1, the mean fasting C-peptide was 3.6 ± 3.6 (0.2–11.8) ng/mL and after glucose stimulation was 5.0 ± 5.0 (2.1–15) ng/mL. These relatively low levels reflect the impoverished insulin secretion of long-standing insulin resistant diabetes and are associated with high-dose exogenous insulin, glucotoxicity, and β-cell failure. We see this same phenomenon in patients with insulin receptor mutations and extreme insulin resistance.

**TABLE 1:**
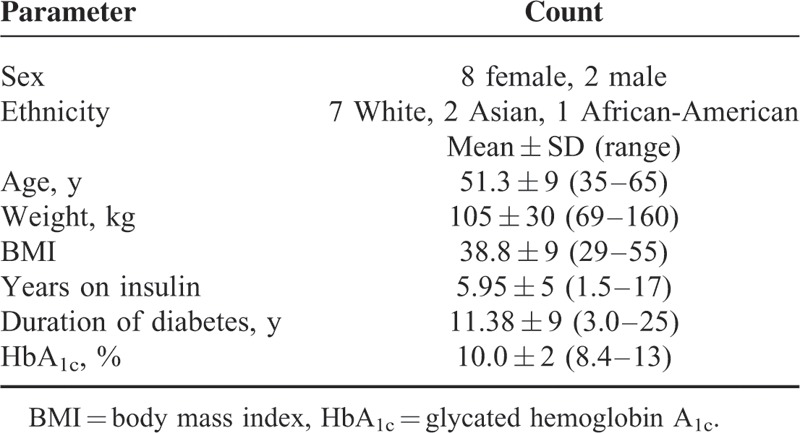
Baseline Characteristics for 10 Patients With Type 2 Diabetes and Severe Insulin Resistance

Upon admission, patients were initiated on the calorically restricted diet. The preadmission caloric needs were estimated to be 2210 ± 371 (1695–3035) kcal/d. The in-hospital caloric intake was 1810 ± 202 (1600–2200) kcal/d. The caloric restriction was on average a 16.5% reduction (400 ± 401 kcal/d) from the calculated preadmission caloric need.

**FIGURE 1:**
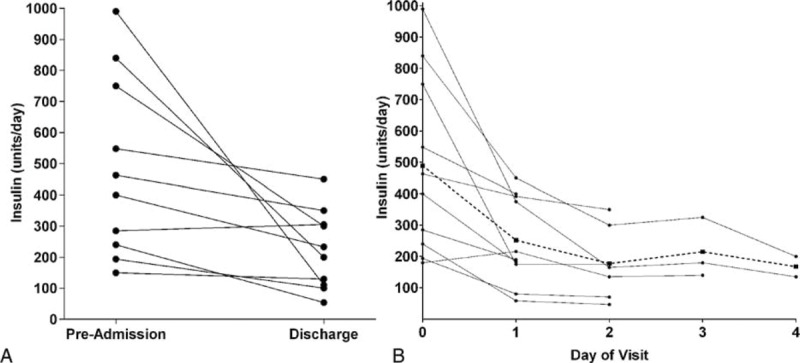
(A) Insulin requirements before admission and after 3 to 6 days of mild caloric restriction in 10 patients with type 2 diabetes and severe insulin resistance. (B) Insulin requirements on each day of the hospitalization in the same patients (solid black lines/circles represent individual patients, dashed line/squares show the mean for all patients).

Figure 1A displays the decrease in insulin dose from preadmission to discharge for each patient. Insulin doses decreased during the intervention in 9 of 10 subjects. The insulin dose decreased by a mean of 44% or 263 ± 296 (−880 to +20) units/d. Additionally, the insulin doses decreased from 486 ± 291 (150–990) units/d preadmission to 223 ± 127 (54–450) units/d at discharge (*P* = 0.02). Insulin dose in units/kg/d decreased from 4.8 ± 3.1 (1.2–9.6) to 2.2 ± 1.3 (0.6–4.5). Repeated measures analyses showed that insulin doses significantly decreased over the hospitalization (*P* = 0.0025), with the greatest change in insulin requirement occurring between preadmission and hospital day 1 (Figure 1B).

**FIGURE 2:**
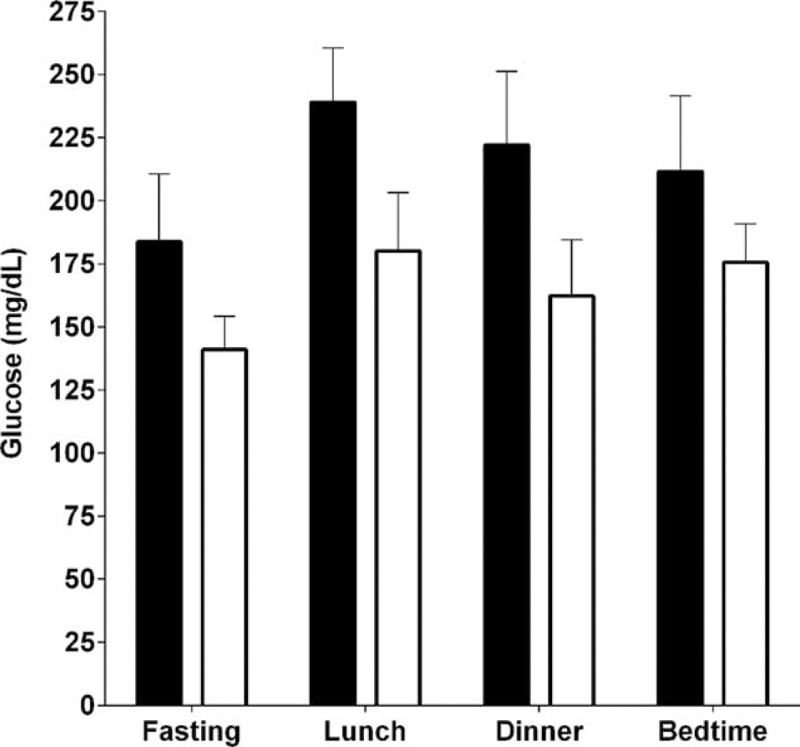
Mean (SD) capillary blood glucose in the first 24 hours after admission (black bars) versus after 3 to 6 days of mild caloric restriction (white bars) in 10 patients with type 2 diabetes and severe insulin resistance in the fasting state, prelunch, predinner, and at bedtime.

**FIGURE 3:**
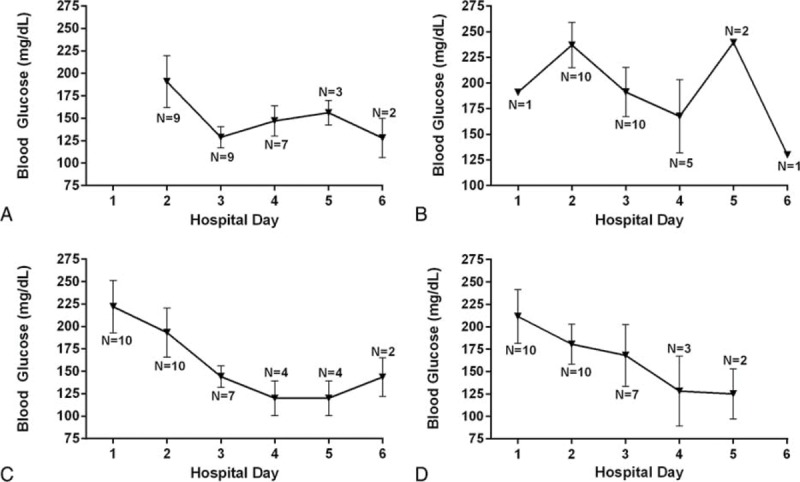
Mean (SD) capillary blood glucose on each day of the hospitalization during 3 to 6 days of mild caloric restriction in 10 patients with type 2 diabetes and severe insulin resistance in the fasting state (A), prelunch (B), predinner (C), and at bedtime (D).

Despite reductions in insulin dose, blood glucose levels remained stable or decreased over the hospital visit. Fasting, lunch, and bedtime glucoses decreased nonsignificantly from the first to last day of the hospital stay (*P* = 0.2, 0.06, and 0.2, respectively), whereas dinner glucose significantly decreased (*P* = 0.02) (Figure 2). Results were similar for repeated measures analyses over the course of the hospitalization (Figure 3).

## DISCUSSION

This study demonstrates the effectiveness of modest caloric restriction in the treatment of SIR. Insulin requirements decreased in 9 of 10 patients within 24 hours as a result of caloric restriction. The observed dose reduction of 44%, or 263 units (as high as 880 units), is highly clinically significant, and is well beyond the reduction that would be anticipated by reduced carbohydrate consumption alone. Although insulin sensitivity was not directly measured in this study, these reductions in insulin dose with stable or declining blood glucose are consistent with rapid improvements in insulin sensitivity, analogous to that observed in more severely calorie restricted states, such as VLCDs or bariatric surgery. An additional explanation for the decreased exogenous insulin requirement is recovery of β-cell function due to reduced glucose toxicity; however, this is less likely given the relatively small changes in blood glucose observed during the study. Furthermore, the basal C-peptide levels are low for this degree of insulin resistance and increased very little under further glucose stimulation.

One of the strengths of this study is that it was conducted in a controlled clinical environment, thus ensuring patient compliance with the intervention. The study was limited by small sample size; however, SIR in T2D remains an uncommon condition despite its growing prevalence. In addition, patients were recruited through physician referral rather than population sampling, potentially biasing the results. This study was also limited by the retrospective nature of the data collection, which required calculation of some prehospital insulin doses based on HbA1c, as well as calculation of prehospital energy intake. Although it is possible that the observed reduction in insulin doses during the hospitalization was due to patient noncompliance with home insulin regimens, this is unlikely as patients with either a history of noncompliance or current noncompliance based on investigator judgment were excluded from the study. There was a variable duration of intervention, as well as variability in the percent caloric restriction. Although weight was not measured on a daily basis during the hospital stay, given the short duration and modest dietary restriction, it is reasonable to assume that body weight did not change substantially.

Unfortunately, we were unable to collect data on the longer-term efficacy of the diet intervention after subjects were discharged. We postulate that the modest caloric restriction should be sustainable outside a hospital environment, but it is possible that patients may return to their preadmission caloric intake. Moreover, it is unclear if these dramatic effects on insulin requirements would be sustained only during a period of acute negative energy balance, or if the benefits would continue after weight stabilization and equilibration at the new caloric intake level. There is a suggestion that these effects might continue.^12^ Further studies with outpatient follow-up are needed to determine the duration of efficacy as well as patient compliance to the diet intervention.

In conclusion, there is very little published on how to effectively manage nonsyndromic patients with T2D and SIR. This study suggests that very modest caloric restriction may substantially reduce insulin requirements in this population. Although following a low carbohydrate/low calorie diet is a key part in managing T2D and insulin resistance, it is often overlooked or its importance underestimated by the patients and not reinforced enough by medical providers. These reductions in insulin doses may serve to motivate patients regarding both medication and dietary compliance, as they gain the confidence to know that their diabetes can be effectively managed. Furthermore, this intervention is low cost and accessible to all patients. In every diabetic patient the diet is in fact the key for success. Thus, the role of diet, while different to achieve, cannot be overestimated. Further research should be done to fully understand the importance of this potential therapy for SIR.
